# Physician and patient perspectives on hypertension management and factors associated with lifestyle modifications in Japan: results from an online survey

**DOI:** 10.1038/s41440-020-0398-0

**Published:** 2020-01-29

**Authors:** Nobuhiro Nishigaki, Yukio Shimasaki, Takuo Yoshida, Naoyuki Hasebe

**Affiliations:** 1 Japan Medical Office, Takeda Pharmaceutical Co. Ltd, Tokyo, Japan; 2 Division of Cardiology, Nephrology, Pulmonology and Neurology, Department of Internal Medicine, Asahikawa Medical University, Asahikawa, Hokkaido Japan

**Keywords:** Education, Lifestyle modification, Hypertension paradox, Adherence, Hypertension

## Abstract

We conducted a survey to examine the gaps between Japanese physician and patient perspectives on hypertension management and to investigate important factors that may help solve the “hypertension paradox” in Japan. Web-based surveys of patients and physicians were conducted in Japan between October 19 and 31, 2017. The data collected included physician and patient perspectives on hypertension education, adherence to lifestyle modifications and antihypertensive medication, and reasons for treatment adherence/nonadherence. Factors relating to specific patient behaviors (e.g., monitoring their home blood pressure [BP] daily) were analyzed by multivariate logistic regression analysis. Of the 541 physicians and 881 patients included in the analyses, both groups recognized that the extent of lifestyle changes was insufficient. Approximately 80% of physicians reported that they fully or sufficiently provided education to patients about reasons for hypertension treatment and its associated risks, target BP levels, and lifestyle modifications. Only 40–50% of patients considered those topics having been fully or sufficiently discussed. Logistic regression analyses revealed that positive lifestyle modifications (daily home BP monitoring, salt intake <6 g/day, and daily aerobic exercise for ≥30 min) were positively associated with receiving feedback from physicians about specific lifestyle modifications and patient motivation for maintaining their target BP. In conclusion, perception of the amount of education provided by physicians on hypertension management was lower in patients than in physicians. In addition to effective regular follow-up regarding lifestyle modifications, patient motivation by physicians is an important factor for improving lifestyle modifications and achieving effective hypertension management in Japan.

## Introduction

High blood pressure (BP) is a major risk factor for cardiovascular and renal diseases [1, 2], the leading global risk factor responsible for disability-adjusted life years [3], and the second leading risk factor for adult mortality in Japan [4]. Despite advances in the diagnosis and management of high BP, there continues to be a high prevalence of hypertension worldwide, with an estimated 1 billion patients over the age of 25 diagnosed in 2008 [5]. Similarly, there is a high prevalence of hypertension among the Japanese population [6–8], which had ~43 million patients with hypertension in 2010 [7, 8], which equates to approximately one-third of all adult males and one-quarter of adult females in Japan [9].

Hypertension can be categorized as primary (95% of all hypertension cases) or secondary [10]. Although the causes of primary hypertension are not fully known, it has been attributed to several risk factors that are nonmodifiable (e.g., age, race, and genetic composition) and modifiable (e.g., being overweight/obese, sodium and potassium intake, poor physical fitness, and high alcohol consumption) [11]. Population-based strategies involving lifestyle modifications can be implemented to prevent primary hypertension [12]. Furthermore, decreases in systolic BP at the population level have been associated with substantial decreases in BP-related illnesses such as stroke and cardiovascular disease [12]. Current guidelines issued by the Japanese Society of Hypertension (JSH) recommend lifestyle modifications as the initial treatment for hypertension, including decreased salt intake, weight loss, exercise, and restricted alcohol intake [8]. These lifestyle modifications have been shown to both suppress the onset of hypertension and manage high BP once a patient is diagnosed and treatment has commenced [8, 13]. However, if lifestyle changes alone are not, or are not expected to be, sufficient for adequate control of BP, or if the patient has additional risk factors (e.g., advanced age, obesity, diabetes mellitus, dyslipidemia, or preexisting cardiovascular disease), then antihypertensive medication is also recommended [14]; antihypertensive medications include diuretics, beta-blockers, angiotensin converting enzyme inhibitors, calcium channel blockers, and angiotensin II receptor blockers [8, 13].

Treatment rates among Japanese patients with hypertension have increased from 1980 to 2010 (in 2010, >50% of patients aged ≥60 years were treated), but only ~30% of male and >40% of female patients with hypertension can be considered as having adequately controlled hypertension (i.e., their BP is <140/90 mmHg) [7], a figure that is clearly unsatisfactory. The most common explanations for this failure to control hypertension are insufficient patient lifestyle changes [14] and nonadherence to antihypertensive drugs, as well as treatment-resistant hypertension [15–18]. While knowledge of factors that contribute to nonadherence to lifestyle and therapeutic interventions is limited, it is widely believed that effective patient education (e.g., explanation of hypertension and the aims, effects and adverse effects of treatment, and good physician–patient relationships) is vital to improve BP control rates and achieve better patient quality of life and long-term clinical outcomes [8, 15, 19–21]. The 2017 American College of Cardiology/American Heart Association, 2018 European Society of Cardiology/European Society of Hypertension, and JSH 2014 guidelines recommend team-based behavioral and motivational strategies to achieve healthy lifestyles [8, 13]. Moreover, cognitive-behavioral strategies including goal setting, BP self-monitoring, and adequate and frequent feedback from physicians have been suggested as key interventional components to change behavior regarding physical activity and dietary lifestyle modifications [13, 22].

With these issues in mind, we conducted an online survey (**P**erspectives of P**a**tients and Physicians **R**eg**a**r**d**ing Hypertensive Management from an **O**nline Survey for E**x**cellence; PARADOX study) to gain further insight into why a large proportion of Japanese patients with hypertension do not achieve the target BP levels, as defined by the JSH, despite receiving treatment (the so-called “hypertension paradox”) [8, 23]. Surveys were administered to both physicians and patients to assess similarities and differences in their perspectives on hypertension management, particularly relating to the provision of education and how it may impact adherence to lifestyle changes and antihypertensive medication. Physicians were also asked about their perspectives on hypertension diagnosis practices.

## Methods

### Objective

The objective of the survey was to investigate the reasons behind the low rate of achievement of BP targets in Japanese patients with hypertension. This was examined by investigating the potential similarities and differences between physician and patient perspectives on the diagnosis and/or management of hypertension. We focused on guidance provided or received at consultation and factors related to self-management practices.

### Study design

The PARADOX study was a panel-based, cross-sectional, observational study of physicians and patients in Japan, in which target respondents identified through research panels completed self-administered, web-based surveys. Invited physicians were members of a research panel—Nikkei Medical Online (Tokyo, Japan)—which is a free-to-register portal site with medical information for physicians and healthcare professionals, operated by Nikkei Business Publications, Inc. (Tokyo, Japan). As of October 31, 2017, there were 153,666 member physicians [24], and the target number for this study was ≥500 physicians, of whom 100 had to be from cardiology departments. The invited patients (24,059 in total) comprised members of two survey platforms managed by Research Panel, Inc. (Tokyo, Japan) and GMO Research, Inc. (Tokyo, Japan), and the target number for this study was 1000 patients.

The physician survey comprised nine screening and 23 survey questions, while the patient survey comprised 13 screening and 22 survey questions. All questions were developed by the authors in collaboration with index-i Corporation (Tokyo, Japan) to examine whether differences in perspective on the management of hypertension between patients and physicians led to the “hypertension paradox” in Japan. The questionnaires were reviewed and revised by a cardiologist and a hypertension specialist to validate the content, and the answer options for the multiple choice questions were randomized to eliminate answer option order bias. The survey data included information on demographics, hypertension and comorbidities, duration of consultations, hypertension education and guidance, adherence to lifestyle changes and antihypertensive medication, and factors potentially impacting adherence and treatment success.

Both surveys were administered by index-i Corporation between October 19 and 31, 2017. Physicians and patients were invited by e-mail to participate in the online surveys, which had to be completed during the study period to be included in the study. Sample physician and patient surveys are included in the Supplementary Information.

### Physicians and patients

Physicians and patients from all prefectures in Japan were invited to participate in the study and were screened for eligibility. The physician survey comprised nine screening (Supplementary Document 1) and 23 survey questions (Supplementary Document 2), while the patient survey comprised 13 screening (Supplementary Document 3) and 22 survey questions (Supplementary Document 4). Physicians were eligible to participate if they were ≥24 years of age, had treated ≥30 patients with hypertension in the past month, and could prescribe antihypertensive medication freely according to their own management policy. Patients of both sexes were included if they were aged between 20 and 89 years, were currently prescribed antihypertensive medication, and had been assessed for hypertension in the past year by a physician. Patients without an initial diagnosis of hypertension and those who did not undergo regular follow-up assessments were excluded. The age and sex composition of the analyzed patient population was matched to that of patients with hypertension in Japan, as outlined in the JSH 2014 guidelines [8]; once the target proportion of men and women in the predefined age groups (20, 30, 40, 50, 60, 70, and 80 s) was reached, subsequent respondents were excluded from the analysis.

The collected data were cleaned by excluding respondents providing inconsistent or unrealistic answers to the questions (Supplementary Document 5) before carrying out the analysis. Of the physicians who participated in this study, physicians were excluded from the analysis set if their target systolic BP was <100 or ≥200 mmHg; their target diastolic BP was <60 or ≥140 mmHg; or if the same answer (from seven options available) was selected in response to all questions pertaining to how much the 31 candidate factors contributed to the low rate of achievement of BP targets in patients with hypertension, which suggested that the respondent had not given due thought to their responses (Supplementary Document 5).

Of the patients who participated in this study, patients were excluded from the analysis set if they responded that they were prescribed over 30 types of drugs; their age and years with hypertension were the same; their initial or follow-up consultation was more than 90 min; their systolic BP was <100 mmHg and their diastolic BP was <60 mmHg; their target systolic BP was <100 or ≥200 mmHg and target diastolic BP was <60 or >140 mmHg; and if their responses did not match (e.g., if they reported that they “always share all my home BP records with my physician,” but also answered that they “never record their home BP”) (Supplementary Document 5).

The study was conducted in accordance with the ethical principles of the Declaration of Helsinki and the International Conference on Harmonisation Guideline for Good Clinical Practice. All respondents consented to participate in the surveys and were allowed to discontinue the survey at any time. All responses were anonymized (in an unlinkable manner) at the time of data collection.

### Statistical analysis

The results are presented descriptively as the percentage of physicians/patients responding to a question. Factors relating to “specific patient behaviors” (daily BP monitoring, reducing salt intake to 6 g/day, and undertaking daily aerobic exercise for ≥30 min) were analyzed by multivariate logistic regression analysis adjusted for age, sex, region of residence in Japan, type of institution the patient visited, employment status, and absence or presence of cardiovascular and renal complications in patients recording their home BP and were expressed as odds ratios and 95% confidence intervals (CIs). All education factors examined at the initial and follow-up consultations and all reasons cited by patients for continuing daily home BP measurements were analyzed as candidate factors. The statistical impact of these factors was assessed using a Wald chi-square test at a significance level of *α* = 0.05. Logistic regression analyses were performed by Creativ-Ceutical K.K. (Tokyo, Japan) using SAS version 9.3 (SAS Institute; Cary, NC, USA), under instruction from the authors.

## Results

### Physician and patient demographics

A total of 565 physicians from all prefectures in Japan were surveyed during the study period, and 541 physician responses were included in the analyses. Of these, 24 responses were excluded from the analysis—19 because the respondents gave the same answers to all 31 factors for questions 21 and 22 (Supplementary Document 2 and 6). The physicians who responded to the survey were predominantly male (92.4%), with a mean age of 51.5 years; most practiced within a general hospital (57.1%) or clinic (32.2%); their most common specialties were internal medicine (49.0%) and cardiology (20.3%); and ~1 in 10 of the physicians (10.9%) were JSH certified (Table 1). Physicians reported that two-third of the patients under their care presented with coexisting diseases (half of their patients were ≥75 years of age); most frequently, these diseases were diabetes mellitus, kidney disease, heart disease, and brain diseases (stroke, etc.) (all Table 1).

**Table 1:** Physician demographics

Characteristics	Demographics (*n* = 541)
Age, years	51.5 ± 9.6
Male, *n* (%)	500 (92.4)
Type of institution, *n* (%)	
General hospital	309 (57.1)
Clinic (no beds available)	175 (32.3)
University hospital	40 (7.4)
Clinic (≤19 beds)	17 (3.1)
Specialty, *n* (%)	
Internal medicine	265 (49.0)
Cardiology	110 (20.3)
Neurology	37 (6.8)
Nephrology	33 (6.1)
Diabetes	30 (5.5)
Other	24 (4.4)
Surgery	13 (2.4)
Gastroenterology	13 (2.4)
Respiratory medicine	10 (1.8)
Gerontology	3 (0.6)
Pediatrics	2 (0.4)
Plastic surgery	1 (0.2)
JSH-certified physician, *n* (%)	59 (10.9)
Average length of assessment at:	
Initial diagnosis, minutes	15.6 ± 6.5
Follow-up, minutes	6.6 ± 3.4
Number of patients treated in the last month, median (min, max)	120 (30, 995)
<75 years of age, median (min, max)	60 (0, 660)
≥75 years of age, median (min, max)	60 (0, 540)
Number of patients with comorbidity, median (min, max)	80 (0, 735)
Diabetes, median (min, max)	30 (0, 730)
Kidney disease e.g., CKD, median (min, max)	20 (0, 400)
Heart disease e.g., CAD, median (min, max)	20 (0, 300)
Neurological disorder e.g., CVD, median (min, max)	16 (0, 250)
Other, median (min, max)	0 (0, 500)
Number of patients without comorbidities, median (min, max)	35 (0, 500)

Data shown as mean ± standard deviation unless otherwise stated

*CAD* coronary artery disease, *CKD* chronic kidney disease, *CVD* cerebral vascular disease, *max* maximum, *min* minimum, *JSH* Japanese Society of Hypertension

There were 1170 patients who participated in the survey, and 881 were included in the analysis. Of the 289 responses excluded from the analysis, 201 were excluded to match the age and sex composition of the patient population to that of patients with hypertension in Japan, as outlined in the JSH 2014 guidelines (inclusion of respondents was based on the time of survey completion), and 37 responses were excluded due to a mismatch in their responses to questions 21 and 5 (Supplementary Document 4 and 6). Of these patients, 54.0% were male; the mean age was 62.5 years; and patients had been diagnosed with hypertension for a mean of 9.7 years (Table 2). The most frequently reported comorbidities among patients were dyslipidemia (25.7%) and diabetes mellitus (14.9%); 6.9% of patients reported coexisting heart disease. Most patients stated that they received their care at a clinic (70.9%) or general hospital (24.4%; all Table 2).

**Table 2:** Patient demographics

Characteristics	Demographics (*n* = 881)
Age, years	62.5 ± 13.8
Male, *n* (%)	476 (54.0)
Comorbidities, *n* (%)	
Dyslipidemia	226 (25.7)
Diabetes	131 (14.9)
Hyperuricemia/gout	75 (8.5)
Heart disease^a^	61 (6.9)
Digestive disease^b^	53 (6.0)
Obesity	45 (5.1)
Insomnia	38 (4.3)
Stroke^c^	35 (4.0)
Mental illness^d^	34 (3.9)
Asthma	30 (3.4)
Kidney disease^e^	24 (2.7)
Sleep apnea	17 (1.9)
Vascular disease^f^	10 (1.1)
COPD	8 (0.9)
Liver disease^g^	8 (0.9)
Other	88 (10.0)
Number of years since diagnosis	9.7 ± 8.2
Type of institution where care is primarily received, *n* (%)	
Clinic	625 (70.9)
General hospital	215 (24.4)
University hospital	41 (4.7)
Average length of assessment at:	
Initial diagnosis, minutes (*n* = 446)^h^	17.5 ± 13.2
Follow-up, minutes (*n* = 881)	9.4 ± 8.9

Data shown as mean ± standard deviation unless otherwise stated

*COPD* chronic obstructive pulmonary disease

^a^e.g., angina, myocardial infarction, heart failure

^b^e.g., gastroenteritis, gastric ulcer, ulcerative colitis

^c^e.g., cerebral infarction, cerebral hemorrhage, subarachnoid hemorrhage

^d^e.g., depression, schizophrenia

^e^e.g., chronic kidney disease

^f^e.g., aortic dissection, aortic aneurysm

^g^e.g., cirrhosis

^h^Do not remember, *n* = 435

### Physician perspectives on why the rates of achieving target BP levels are low versus patients’ daily practices of managing hypertension

**Fig. 1:**
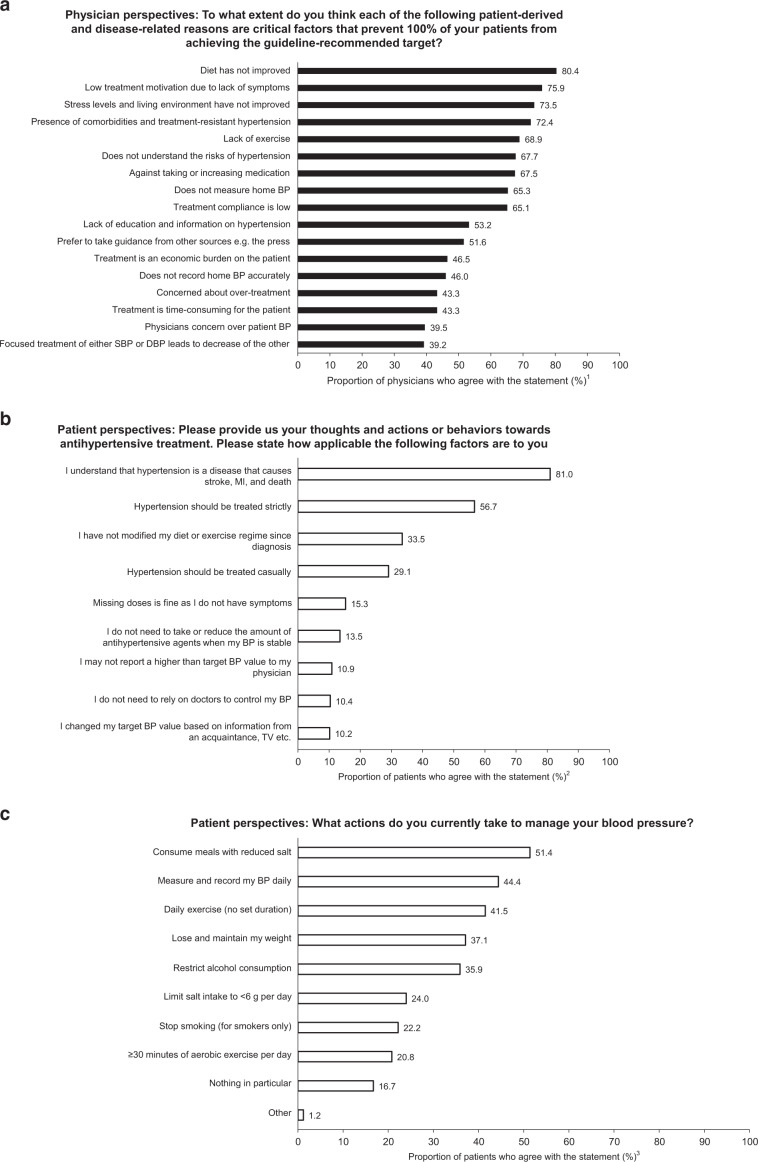
Patient and physician beliefs, attitudes, and behaviors toward hypertension management. **a** Physician-reported factors contributing to patients’ failure to achieve blood pressure targets; **b** patients’ attitudes toward hypertension treatment and their associated behaviors; and **c** patient-reported lifestyle changes for blood pressure management. BP blood pressure, DBP diastolic blood pressure, MI myocardial infarction, SBP systolic blood pressure, TV television. ^1^Percentage of physicians responding in each category with the answers “very significant” or “significant.” Based on physician question Q21 (Supplementary Document 2; to what extent do you think each of the following patient-derived and disease-related reasons are critical factors that prevent 100% of your patients from achieving the guideline-recommended target? Please select the most appropriate response for each of the following factors [scale ranging from “very significant” to “not significant at all”]). ^2^Percentage of patients who answered “very applicable” and “somewhat applicable” for each item. Based on the patient screening question SC13 (Supplementary Document 3; Please provide us your thoughts and actions or behaviors regarding antihypertensive treatment. Please select the most appropriate response for each of the following [scale ranging from “very applicable” to “not at all applicable”]). ^3^Percentage of patients who answered “yes” for each item. Based on patient question Q4 (Supplementary Document 4; what actions do you currently take to manage your blood pressure? Please select all the factors that apply to you from the following options)

According to physicians, the most important factor leading to a low rate of achievement of target BP levels was the lack of an improved diet (80.4%; Fig. 1a). Furthermore, the following factors were also reported as reasons for low hypertension treatment success rates by over 60% of physicians: patients’ failure to implement other lifestyle modifications (reduce stress [73.5%] or increase exercise [68.9%]), lack of therapeutic motivation (75.9%), poor patient self-management (poor adherence to home BP monitoring [65.3%] and drug use [65.1%]), and failure to understand the risks associated with hypertension (67.7%) (Fig. 1a).

Although many physicians believed that poor understanding of hypertension-associated risks was a major cause of the low rates of target BP achievement, 81.0% of patients responded that they knew that hypertension was a disease that causes death (Fig. 1b). In addition, 56.6% of patients responded that hypertension should be treated strictly (Fig. 1b). However, 33.5% reported no change in their diet or exercise regimen since their diagnosis, and 29.1% believed that hypertension could be treated casually (Fig. 1b). Between 10 and 15% of patients responded that they felt no need to take their antihypertensive medication as they were asymptomatic or when their BP had stabilized; there was no need to notify their physician even if their BP was higher than the target level; they could control their BP themselves without relying on their doctor; and they had changed their target BP values based on information provided by an acquaintance or seen on TV, etc. (Fig. 1b).

Self-management to control BP, such as home BP measurement, lifestyle modifications, and treatment adherence, are key to the successful treatment of hypertension [8, 13]. Even the most frequent lifestyle changes reported by patients for managing their BP were executed by ~50% of patients. These included consuming meals with a reduced salt content (51.4%), followed by recording daily home BP in a diary (44.4%), exercising daily with no fixed duration (41.5%), losing and subsequently maintaining weight (37.1%), and restricting alcohol intake (35.9%; all Fig. 1c). Notably, <25% of patients modified their lifestyle to keep their salt intake to <6 g/day (24.0%) and to perform aerobic exercise for more than 30 min a day (20.8%; all Fig. 1c).

### Hypertension education and guidance at initial and follow-up consultations

**Fig. 2:**
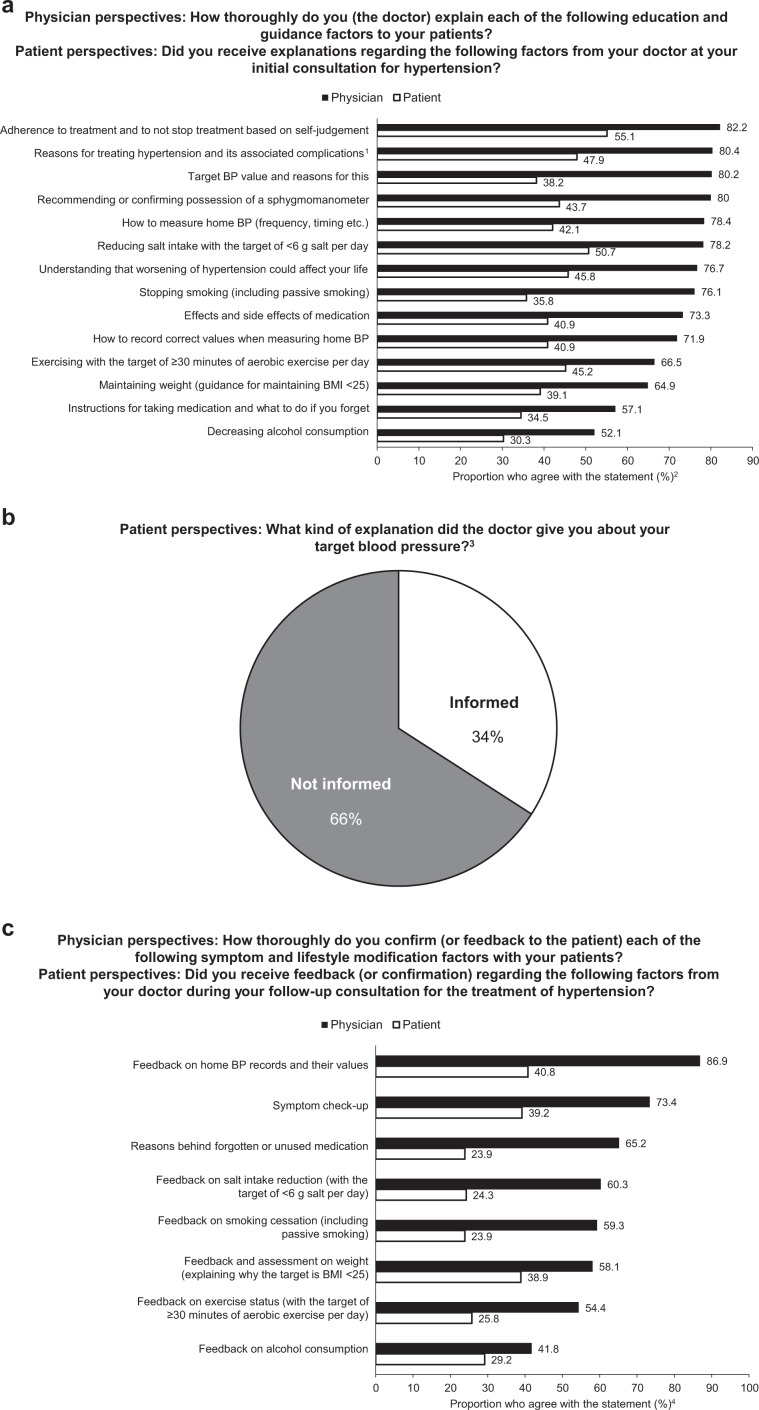
Physician and patient perspectives on the education and guidance on hypertension and its management. **a** Physician and patient perspectives on the education and guidance provided at the initial diagnosis, **b** patient-reported education regarding target blood pressure, and **c** physician and patient perspectives on the feedback or confirmations attained during follow-up assessments. BP blood pressure, BMI body mass index, DBP diastolic blood pressure, SBP systolic blood pressure. ^1^Chronic kidney disease, myocardial infarction, stroke, etc. ^2^Percentage of patients and physicians who responded “firmly” or “to some extent.” Based on patient question Q2 (Supplementary Document 4; did you receive explanations regarding the following factors from your doctor at your initial consultation for hypertension? Please select the most appropriate response for each factor, and how thoroughly you received explanations from your doctor) and physician question Q3 (Supplementary Document 2; how thoroughly do you (the doctor) explain each of the following education and guidance factors to your patients? Please select the most appropriate response that applies to each patient education factor). Scale ranging from “very thoroughly” to “none” for both questions. ^3^Based on patient question Q11 (Supplementary Document 4; what kind of explanation did the doctor give you about your target blood pressure? Please provide the numerical target value if your doctor provided you with one). Figure depicts the sum of responses in which the patients reported “I was informed of the numerical value” and “I was informed of the numerical range.” Respondents were asked whether a specific value or a range was provided for both SBP and DBP. ^4^Based on patient question Q3 (Supplementary Document 4; did you receive feedback (or confirmation) regarding the following factors from your doctor during your follow-up consultation for the treatment of hypertension? Please select the most appropriate response for each factor, regarding how thoroughly you received feedback (or confirmation) from your doctor) and physician question Q5 (Supplementary Document 2; how thoroughly do you confirm (or feedback to the patient) each of the following symptom and lifestyle modification factors with your patients? Please select the most appropriate response that applies to each patient education factor). Scale ranging from “very thoroughly” to “none” for both questions

The duration of initial consultation was similar according to physicians (mean: 15.6 ± 6.5 min; Table 1) and patients (17.5 ± 13.2 min; Table 2). Overall, physicians reported that they had fully or sufficiently provided education and guidance for all factors, including the consequences of hypertension, the BP targets, the importance of home BP measurements, how to measure and record home BP, the effects and adverse effects of medication, and the need for lifestyle modification and medication (range, 52.1–82.2%; Fig. 2a), however, a lower proportion of patients reported that they had received full or sufficient education and guidance (range, 30.3–55.1%; Fig. 2a), at initial consultation. Both physicians and patients agreed that the least guidance was provided for the need to reduce alcohol consumption and the most guidance was provided for the need to continue antihypertensive treatment and to not stop based on self-judgment (Fig. 2a). Furthermore, over 75% of physicians reported that they had fully or sufficiently explained the reasons for hypertension treatment, the associated risks, the target BP levels, the need to stop smoking, how to measure BP at home, how worsening hypertension could affect a patient’s life, and confirmed or suggested that their patients had a sphygmomanometer. In contrast, there were only two factors whereby over 50% of patients felt that full or sufficient education and guidance was provided (the importance of adhering to antihypertensive treatment and not stopping based on self-judgment [55.1%] and the need to reduce salt intake to <6 g/day [50.7%]; Fig. 2a). Notably, 66% of patients were not informed of a specific target BP value or range by their physician (Fig. 2b).

The duration of follow-up consultation was also similar according to physicians (mean: 6.6 ± 3.4 min; Table 1) and patients (9.4 ± 8.9 min; Table 2) and shorter in length than the initial consultation. At the follow-up consultation, physicians reported that they fully or sufficiently assessed patients’ home BP diaries (86.9%) and examined any remaining and/or missed medication (including establishing the causes/reasons for nonadherence; 65.2%), and ~42–60% stated that they examined lifestyle changes (weight, low-salt diet, exercise, alcohol intake, and smoking) fully or sufficiently (Fig. 2c). However, acknowledgment was substantially lower from the patients’ perspective, with less than half of patients reporting that physicians fully or sufficiently assessed their home BP diaries (40.8%), weight (38.9%), alcohol consumption (29.2%), exercise levels (25.8%), salt consumption (24.3%), smoking (23.9%), and any remaining and/or missed medication (23.9%) (Fig. 2c).

### Patient self-management and motivation

**Fig. 3:**
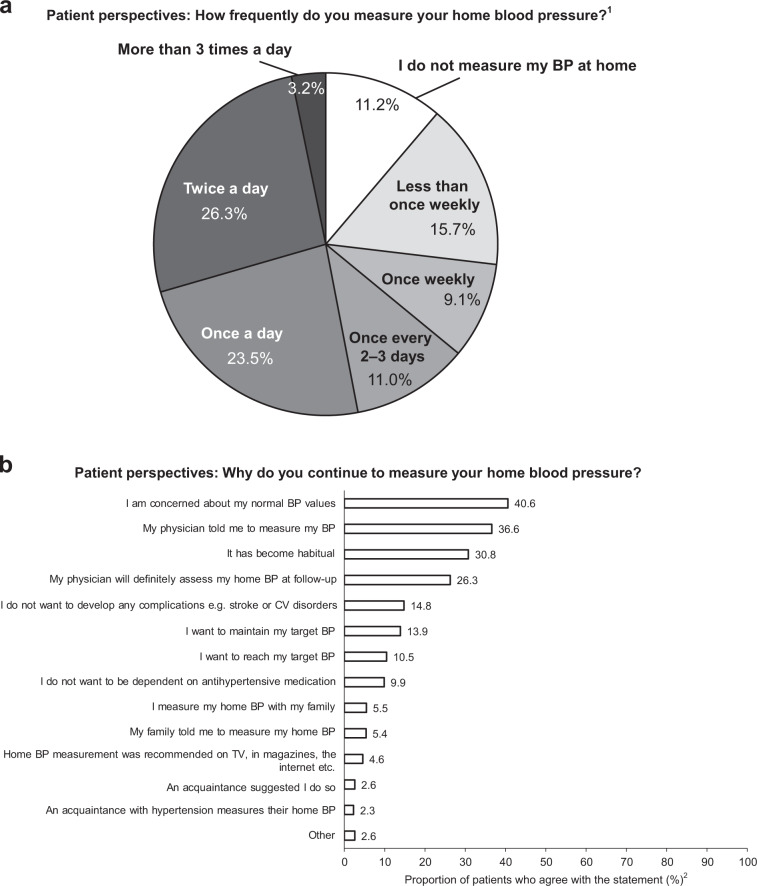
Patient self-management. **a** Patient-reported frequency of home blood pressure measurement and **b** reasons cited by patients for continuing home blood pressure measurement. BP blood pressure, CV cardiovascular, TV television. ^1^Based on patient question Q14 (Supplementary Document 4; how frequently do you measure your home blood pressure? Please choose the most appropriate out of the following options). Respondents chose from the seven options provided. ^2^Percentage of patients who answered “very applicable” and “somewhat applicable” for each item. Based on patient question Q16 (Supplementary Document 4; why do you continue to measure your home blood pressure?)

Most patients who participated in the survey owned a device to monitor their BP at home (90.8%), with 7.4% responding that they did not and 1.8% responding that they did not know. When patients who owned or did not know whether they owned a sphygmomanometer were asked about how often they measured their BP at home, 53.0% responded that they measured it at least every day (23.5% about once daily, 26.3% about twice daily, and 3.2% more than three times daily), 11.0% about every 2–3 days, 24.8% less frequently, and 11.2% did not measure it at all (Fig. 3a). Of the patients who reported that they measured their home BP daily, the most common reasons for continuing home BP measurement were because they were concerned about their BP value (40.6%), they were instructed to measure their BP by their physician (36.6%), measurement had become habitual (30.8%), and because it would be assessed by their doctor at the follow-up consultation (26.3%; all Fig. 3b).

Patient self-monitoring is a key component of cognitive-behavioral strategy. Therefore, we explored the relationship between patient self-monitoring factors, such as patients who “measured their home BP daily”, and candidate factors that may be driving these patient characteristics, including the level of education and guidance received by their physicians at the initial and follow-up consultations, as well as the reasons for continuing daily home BP measurement, via logistic regression analysis.

**Fig. 4:**
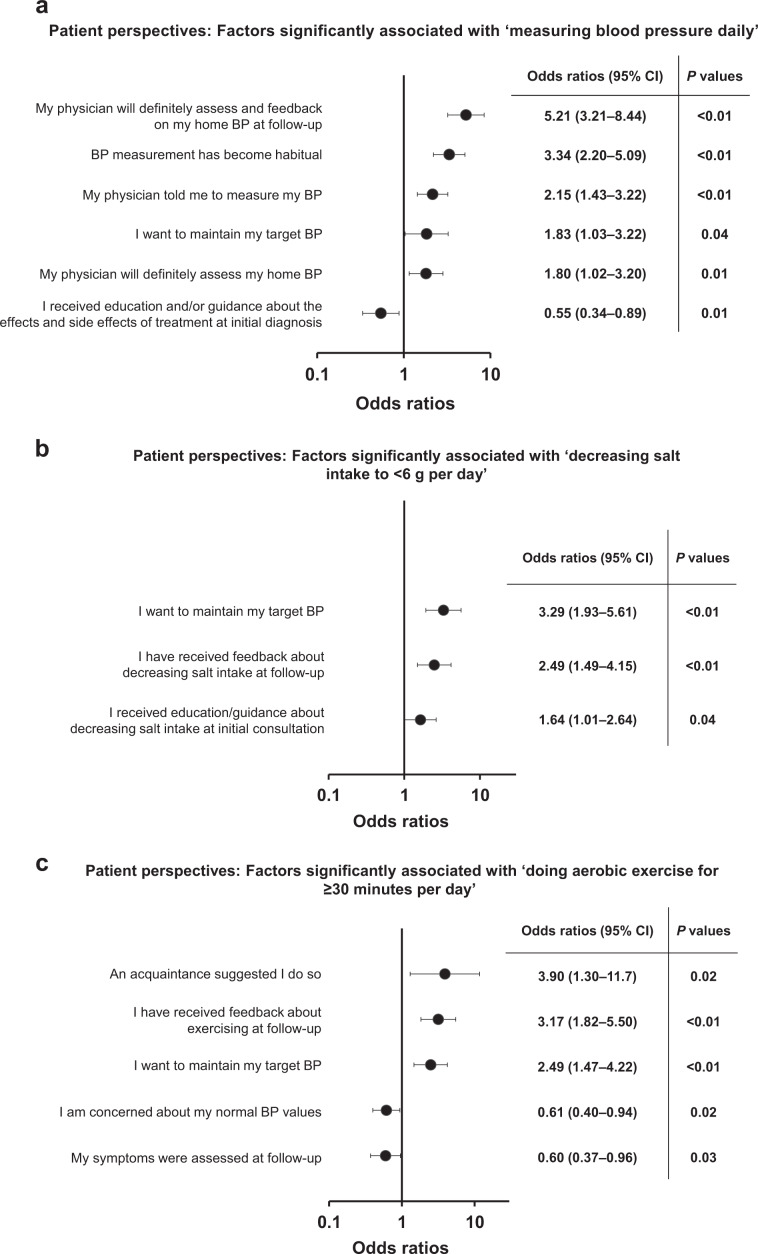
Logistic regression analysis of factors relating to specific patient behaviors. **a** Measuring blood pressure daily, **b** decreasing salt intake to <6 g/day, and **c** undertaking aerobic exercise for ≥30 min per day. BP blood pressure, CI confidence interval. Multivariate logistic regression analysis adjusted for age, sex, region of residence in Japan, type of institution patient visited, employment status, and absence or presence of cardiovascular and renal complications in patients recording their home BP

There was a significant positive association between patients who “measured their home BP daily” and BP confirmation and/or feedback by their physician at the follow-up consultation (OR [95% CI] 5.21 [3.21, 8.44]; *P* < 0.01; Fig. 4a). Significant positive associations were also observed in patients who “measured their home BP daily” with habitual measurement (*P* < 0.01), physician’s instruction to measure BP (*P* < 0.01), BP assessment by physician (*P* = 0.01), and motivation to maintain target BP values (*P* = 0.04; all Fig. 4a). In contrast, a significant negative association was observed between patients who “measured their home BP daily” and patients who received explanation about the effects of treatment and side effects at the initial consultation (OR [95% CI] 0.55 [0.34, 0.88]; *P* < 0.01; Fig. 4a).

Next, we further examined important factors linked to lifestyle modifications that were insufficiently altered by the patient, such as “maintaining salt intake at <6 g per day” and “engaging in aerobic exercise for ≥30 min per day” (Fig. 1c). Logistic regression analyses showed significant positive associations in patients who “maintained their salt intake at <6 g per day” with those motivated to maintain target BP values (OR [95% CI] 3.29 [1.93, 5.62]; *P* < 0.01), those who received feedback on salt intake during follow-up consultations (*P* < 0.01), and those who received guidance at initial consultation for reducing salt intake (*P* = 0.04; all Fig. 4b). Furthermore, significant positive associations were observed in patients who “engaged in aerobic exercise for ≥30 min every day” with those who were recommended to do so by an acquaintance (*P* < 0.02), those who received confirmation regarding their exercise status at their follow-up consultation (*P* < 0.01), and those who were motivated to maintain target BP values (*P* < 0.01; all Fig. 4c). However, significant negative associations were demonstrated between patients who “engaged in aerobic exercise for ≥30 min every day” and those who were concerned about their normal BP (*P* = 0.02) and symptom assessment at the follow-up consultation (*P* = 0.03; all Fig. 4c).

## Discussion

In this survey, we aimed to examine the underlying reasons for the low rate of achievement of target BP levels in Japanese patients with hypertension and how this could be improved by asking physicians and patients about their perspectives regarding the diagnosis and/or management of hypertension. Overall, our study revealed that the education and guidance on lifestyle changes and target BP provided by physicians were not received by patients as much as physicians believed. Moreover, we identified physician-provided assessment and feedback about particular lifestyle modifications and patient motivation for maintaining their target BP as key drivers for the lack of major lifestyle modifications.

Physicians agreed that lifestyle modifications were insufficient in patients with hypertension, with a lack of diet modification (80.4% agreement; Fig. 1a) and exercise (68.9%) being major causes. This is corroborated by the patients’ responses, in which 33.5% of patients reported that they had not changed their diet or exercise regime since receiving a diagnosis (mean number of years since diagnosis: 9.7 years), and is reflective of previous findings [14]. Although 51.4% of patients reportedly consumed meals with reduced salt, only one in four patients actively tried to consume <6 g/day of salt; furthermore, only one in five patients actively exercised for more than 30 min daily and had stopped smoking (for smokers) as part of their life modification therapy. Efforts to promote lifestyle modification in Japanese patients are needed.

Although many physicians (67.7%) believe that the inadequacy of lifestyle modification may be attributed to a lack of understanding around the risks of hypertension, many patients understood the risks of having high BP (81.0%), suggesting that there are clearly other barriers to behavioral change involved. Possible barriers may be that hypertension is generally asymptomatic or that changes in patients with hypertension occur in such slow increments that patients accept these changes as normal (so-called “creeping normality”). There may also be misunderstandings around the importance of making effective and lasting lifestyle modifications.

Accordingly, effective patient education has been suggested to improve the implementation of lifestyle modification in patients with hypertension [8, 13, 22]. In our analysis, physicians responded that they fully or sufficiently provided guidance for hypertension management at the initial diagnosis; however, this education was not fully recognized by patients. In general, patients’ evaluation of the degree and quality of education and/or guidance they received at the initial consultation was ~50–60% of what physicians considered they had provided. These results were similar to a recent study examining physician and patient perspectives in Japan [25], where 86% of physicians and only 39% of patients reported that the aims of treatment were described to them at the initial consultation [25]. Furthermore, the proportion of patients reporting that they received lifestyle management education and guidance was substantially less than what physicians reported providing.

The disparity observed between physician and patient perspectives at the initial consultation was similar to that observed for the regular follow-up assessments, whereby more physicians than patients responded that patient symptoms were checked (73.4% vs. 39.2% in patient responses) and home BP records (86.9% vs. 40.8%) and lifestyle changes (range, 41.8–60.3% vs. 23.9–38.9%) were assessed. Consistent with previous reports, the education and guidance on lifestyle changes and target BP were not understood by patients as much as physicians believed [8, 13, 26, 27]. Physician and patient perspectives regarding initial and follow-up consultations suggest that there is a disconnect between what physicians think they have provided (e.g., the importance of lifestyle changes, adherence to prescribed antihypertensive medication, and regular home BP monitoring and feedback) and what patients actually take away from these meetings. One possible explanation for this disconnect may be that patients do not perceive that they were provided education and/or guidance fully or sufficiently if they did not adequately understand the education and/or guidance [27]. For example, patient literacy level has been linked to patient’s health behaviors [26, 28] and outcomes [29]; thus, a more tailored patient-centric educational model, which takes into account factors such as literacy levels and learning preferences, could yield improved patient outcomes. Considering the additional time that would be required for such patient-centric education, this approach may be achievable with team-based care involving other caregivers, such as nurses and pharmacists, brief counseling sessions on lifestyle with the use of smartphone applications, and cooperation with family members [8, 13, 30].

Cognitive-behavioral change strategies are an essential intervention tool for changing patient behavior [12, 13, 22]. The results from the current analysis show that measuring home BP daily, maintaining salt intake of <6 g/day, and exercising for ≥30 min daily were positively correlated with physician feedback, particularly during follow-up versus initial consultations. This is in agreement with key factors (e.g., prolonged and frequent patient–physician contact and feedback) that are part of cognitive-behavioral strategies [8, 11, 31]. In addition, positive correlations were found between the aforementioned lifestyle modifications and patients’ motivation to maintain their target BP value, which may support the power of motivational interviewing [22]. Home BP measurements are central to self-monitoring of hypertension, and of the patients surveyed [32], 90.4% owned a home BP-measuring device. However, similar to a previous analysis examining the frequency of daily home BP measurements in Japanese patients with hypertension [33], just over half (53.0%) of patients measured their home BP daily. Daily BP measurements showed a trend toward a positive association with exercising for ≥30 min daily (*P* = 0.07) and with maintaining salt intake to <6 g/day (*P* = 0.053). These data may suggest that self-monitoring is effective in promoting lifestyle modification.

Goal setting is a key factor in cognitive-behavioral strategies [22]. In this study, similar to previous studies [25, 34, 35], although most physicians reported that they provided a BP target to their patients, 66% of patients reported that they did not receive a BP target value or range. In agreement with the JSH 2014 guidelines [8], these results suggest that improvements in clear and continuous communication of a target BP to patients combined, if possible, with other patient-centric motivational and cognitive intervention strategies might effectively lead patients to measure their BP daily and make important lifestyle modifications [22].

Feedback from physicians on behavioral changes can provide patients with a measure of and direction for their behavioral changes (e.g., lifestyle modifications to reach a goal, such as a target BP) [8, 13, 22]. Previous studies suggest that feedback and reinforcement result in positive lifestyle modifications [22]. In line with these results, our logistic regression analyses demonstrated that all examined lifestyle modification factors (measuring BP daily, decreasing salt intake to <6 g/day, and exercising for ≥30 min/day) were significantly and positively correlated with feedback at follow-up consultations (all *P* < 0.001). Notably, the influence on patient behavior was larger at the time of follow-up versus at the initial consultation—highlighting the importance of constant feedback as well as physician–patient communication and education and cognitive-behavioral strategies [8, 13, 22].

One key limitation of this study was that improvements in patient lifestyle modification were self-declared. Future studies are required to determine how improvements in lifestyle eventually lead to BP changes. Another limitation is the survey design; as an uncontrolled, observational study, this study could have been subject to potential selection/sampling and response bias. Given that the majority of physicians in Japan treat <30 patients with hypertension per month, restricting the survey to physicians who treat ≥30 patients with hypertension limits the generalizability of the results of this survey. Furthermore, patients and physicians were not matched (i.e., when patients completed the survey, their treating physician was not then queried to also provide a response), which may have led to a mismatch in the results (e.g., the group of physicians surveyed may have been particularly good at providing education and their patients may have had a better opinion of the care provided versus the patient group may have had a different physician group that provided less effective education). Further studies in matched groups of physicians and patients are required to clarify whether the level of education provided to the patients is perceived differently between the physicians and the patients. Finally, the present study does not account for the different categories attributed to the physicians. A subanalysis of the PARADOX study investigating the physicians’ perspective by specialty, institution type, sex, and age has been published [36].

In conclusion, our study shows that, in addition to the provision of effective education and regular follow-up by physicians regarding lifestyle modifications, patient motivation to maintain a target BP is considered an important factor in the achievement of BP goals in Japan.

## Supplementary information

Supplementary Document 1

Supplementary Document 2

Supplementary Document 3

Supplementary Document 4

Supplementary Document 5

Supplementary Document 6
