# Supplementary material for: Physician and patient perspectives on hypertension management and factors associated with lifestyle modifications in Japan: results from an online survey
Source: Hypertens Res. 2020 Jan 29;43(5):450–62. doi: 10.1038/s41440-020-0398-0 (PMC8076050; doi:10.1038/s41440-020-0398-0)
Supplement: Supplementary file 1 — Supplementary Document 1 [file 41440_2020_398_MOESM1_ESM.docx]

**Supplementary Document 1**

**Survey on the Management of Hypertension [Physician]**

**Screening Questions**

SC1. Based on your medical records, please tell us the number of patients to whom you prescribed antihypertensive drugs in the last month.

SC2. When prescribing antihypertensive drugs to patients with high blood pressure, do you make decisions on your own?

SC3. Which type of medical institution do you work at? If you work at multiple institutions, please provide an answer for your main place of work.

SC4. Which medical department are you affiliated with? If you are affiliated with multiple departments, please provide the department in which you mainly treat hypertension.

SC5. Are you a specialist doctor certified by the Japanese Society of Hypertension?

SC6. How long was your average consultation with your patient at initial consultation (first visit) and the subsequent visits (follow-up or regular visits) for management of hypertension?

SC7. What is your gender?

SC8. What is your current age?

SC9. In which prefecture do you mainly work in? If you work at multiple institutions, please provide an answer for your main place of work.
