# Supplementary material for: Physician and patient perspectives on hypertension management and factors associated with lifestyle modifications in Japan: results from an online survey
Source: Hypertens Res. 2020 Jan 29;43(5):450–62. doi: 10.1038/s41440-020-0398-0 (PMC8076050; doi:10.1038/s41440-020-0398-0)
Supplement: Supplementary file 2 — Supplementary Document 2 [file 41440_2020_398_MOESM2_ESM.docx]

**Supplementary Document 2**

**Survey on the Management of Hypertension [Physician]**

**Main Survey**

In this questionnaire, we would like to ask you about how you diagnose and manage hypertension in your practice.

Please tell us about **your patients with hypertension**.

Q1. Based on your medical records, please tell us the number of patients to whom you prescribed antihypertensive drugs in the last month by the categories below.

1. Number of patients by age group (≥75 and <75 years of age)
2. Number of patients by complications or comorbidities (i.e. renal diseases, heart diseases, brain diseases, diabetes mellitus, other, and none)

Q2. Please tell us about your experience in patients with hypertension-related complications.

Please select the most appropriate response for each complication.

Please tell us about the **initial consultation you have with your patients with hypertension**.

Q3. How thoroughly do **you (the doctor)** explain each of the following education and guidance factors to your patients?

Please select the most appropriate response that applies to each patient education factor.

※Responses were selected from 5 choices ranging from ‘very thoroughly’ to ‘none’

[The following question only applies if any educational factor is ‘not explained fully or not explained’ by the physician in Q3]

Q4. In the previous question, you responded that the following education factors were not explained fully to the patient. Please select the education factors that you ask **medical professionals other than yourself (e.g. nurses, pharmacists, etc.)** to explain to the patient.

Please tell us about the **follow-up consultations you have with your patients with hypertension**.

Q5. How thoroughly do you **confirm (or feedback to the patient)** each of the following symptom and lifestyle modification factors with your patients?
Please select the most appropriate response that applies to each patient education factor.

※Responses were selected from 5 choices ranging from ‘very thoroughly’ to ‘none’

Q6. Which **blood pressure value do you use** to make treatment decisions at follow-up consultations?

Please tell us about how you examine the **home blood pressure practices** in your patients with hypertension.

Q7. What proportion (percentage) of your **patients measure home blood pressure**?

Q8. How do you provide guidance to your patients about **when to measure home blood pressure**? Please choose from the following options **what you actually tell your patient**.

Q9. Do you tell your patients how many times to measure their home blood pressure (e.g. attempt twice per measurement)?

[The following question only applies to those who answered multiple attempts per measurement in Q9]

Q10. If your patient measures their home blood pressure more than once, which value do you usually tell your patient to record?

Please tell us how **satisfied you are with the current management of hypertension**.

Q11. How satisfied are you with the **current** **management of hypertension**?

[The following question only applies to those who answered with 1–4 on the satisfaction scale (i.e. those who were not dissatisfied with the current management of hypertension) in Q11]

Q12. In the previous question, you responded that you are satisfied or have no complaints overall with the current management of hypertension; but are there any factors that you are **not satisfied with, or feel you have not yet resolved** regarding the management of patients with hypertension? Please select what applies.

[The following question only applies to those who answered with 5–7 on the satisfaction scale (i.e. those who were dissatisfied with the current management of hypertension) in Q11]

Q12. In the previous question, you responded that you are dissatisfied with the current management of hypertension. Please tell us what you are **not satisfied with, or feel you have not yet resolved,** with regarding the management of hypertension.

Please tell us about the **target blood pressure value in patients with hypertension**.

Q13. For the following example cases, **regardless of the blood pressure reduction target value recommended in the Japanese guidelines**, please tell us the target blood pressure value you set for these patients. Please provide the numerical value of [For Q6, if the respondent answered 1–4, ‘home blood pressure’; if 5 ‘blood pressure at consultation’; or if 6 ‘24-hour blood pressure’] that you usually use as a criterion.

What are **the criteria for strengthening (increasing, combining, or switching) treatment** in patients with hypertension?

Q14. In "young, middle-aged, and older patients (>75 years of age) without complications", at what blood pressure threshold do you decide **to strengthen (increase, combine, or switch) treatment**? Please provide the numerical value of [For Q6, if the respondent answered 1–4, "home blood pressure"; if 5 “blood pressure at consultation”, and if 6 "24-hour blood pressure"] that you usually use as a criterion.

Q15. In response to the blood pressure level you provided in Q14 [the result of Q14 is displayed as “Systolic blood pressure: ● ● mmHg or more; diastolic blood pressure: ● ● mmHg or more.”], what conditions will make you treat the patient more intensively (increase, combine, or switch treatment)?

Q16. Please let us know your thoughts and actions regarding the guidelines?

Q17. Do you think we should aim to achieve the guideline-recommended target blood pressure values, 100% of the time, in clinical practice? Please select from the following options (scale ranging from ‘I strongly agree’ to ‘I do not agree at all’).

[The following question only applies to those who answered any of the options other than ‘I strongly agree’ or ‘I agree’ in Q17]

Q18. Why did you not answer "I strongly agree." or "I agree." about the idea of aiming to achieve the guideline-recommended target blood pressure values 100% of the time in clinical practice? Please select the most appropriate response from the following options.

Q19. Please tell us the percentage of your patients who achieve target blood pressure in each of the following cases.

1. Percentage of your patients achieving guideline-recommended target blood pressure
2. Percentage of your patients achieving your target blood pressure

[The following question only applies to those who answered that they did not achieve target blood pressure in 100% of their patients in 1. of Q19]

Q20. You mentioned that not all (i.e. 100%) of your patients do not achieve the guideline-recommended target blood pressure. From the following options, please select the patient groups who do not achieve this target.

[The following question only applies to those who answered that they did not achieve target blood pressure in 100% of their patients in 1. of Q19]

Q21. To what extent do you think each of the following **patient-derived and disease-related reasons** are critical factors that **prevent 100% of your patients from achieving the guideline-recommended target?**

Please select the most appropriate response for each of the following factors (scale ranging from ‘very significant’ to ‘not significant at all’).

[The following question only applies to those who answered that they did not achieve target blood pressure in 100% of their patients in 1. of Q19]

Q22. To what extent do you think each of the following **pharmacist- and physician-derived reasons** are critical factors that prevent 100% of patients from achieving this target?

Please select the most appropriate response for each of the following factors.

[The following question only applies to those who answered ‘very significant’, ‘significant’ or ‘quite significant’ in any of the factors in Q21]

Q23. In the previous question, you selected the following patient-derived variables as important factors preventing 100% of your patients achieving the guideline-recommended target blood pressure. In order **to resolve these patient-derived factors**, which actions do you wish to increase efforts in more firmly in the future? Please select from the following options (please select a maximum of three options).
