# Supplementary material for: Physician and patient perspectives on hypertension management and factors associated with lifestyle modifications in Japan: results from an online survey
Source: Hypertens Res. 2020 Jan 29;43(5):450–62. doi: 10.1038/s41440-020-0398-0 (PMC8076050; doi:10.1038/s41440-020-0398-0)
Supplement: Supplementary file 3 — Supplementary Document 3 [file 41440_2020_398_MOESM3_ESM.docx]

**Supplementary Document 3**

**Survey on the Management of Hypertension [Patient]**

**Screening Questions**

SC1. Please tell us all diseases or symptoms, which you are receiving treatment for at clinics/hospitals.

SC2. How many medicines are you prescribed in total from clinics/hospitals?

SC3. What is your gender?

SC4. What is your current age?

SC5. What is your occupation?

SC6. Which prefecture do you live in?

Please tell us about **hypertension**.

SC7. How many years have you been attending clinics/hospitals for the treatment of hypertension? If there was a period during which treatment was discontinued, please subtract that period.

SC8. Have you received any antihypertensive drugs in the last year?

SC9. What kind of medical institution do you receive treatment for hypertension? Please select only one from the options (University hospital, General hospital, Clinic).

SC10. How long was your average consultation with your physician at initial consultation (first visit) and the subsequent visits (follow-up or regular visits) for treatment of hypertension? Please select “I do not remember” if you do not remember. Please exclude the time spent on clinical examinations, and only include time spent talking with your doctor.

Please tell us about **information for hypertension and the treatment of hypertension**.

SC11. What is the source of information for the treatment of hypertension, which you refer to or are impacted by? Please select (a) all applicable options, and (b) the most applicable one.

SC12. What kind of information regarding hypertension would you like to have, but feel you do not have adequate access to? Please select all applicable options.

SC13. Please provide us your thoughts and actions or behaviors towards antihypertensive treatment. Please select the most appropriate response for each of the following [scale ranging from ‘very applicable’ to ‘not at all applicable’]).
