# Supplementary material for: Physician and patient perspectives on hypertension management and factors associated with lifestyle modifications in Japan: results from an online survey
Source: Hypertens Res. 2020 Jan 29;43(5):450–62. doi: 10.1038/s41440-020-0398-0 (PMC8076050; doi:10.1038/s41440-020-0398-0)
Supplement: Supplementary file 4 — Supplementary Document 4 [file 41440_2020_398_MOESM4_ESM.docx]

**Supplementary Document 4**

**Survey on the Management of Hypertension [Patient]**

**Main Survey**

In this questionnaire, we would like to ask you about the treatment you receive for hypertension.

Please tell us about the **first time you visited a clinic/hospital for the treatment of hypertension.**

.

Q1. Please select the factors that prompted you to visit a clinic/hospital for the first time.

Q2. Did you receive explanations regarding the following factors from your doctor at your initial consultation for hypertension? Please select the most appropriate response for each factor, and how thoroughly you received explanations from your doctor.

※Responses were selected from 5 choices ranging from ‘very thoroughly’ to ‘none’

Q3. Did you receive feedback (or confirmation) regarding the following factors from your doctor during your follow-up consultation for the treatment of hypertension? Please select the most appropriate response for each factor, and how thoroughly you received feedback (or confirmation) from your doctor.

※Responses were selected from 5 choices ranging from ‘very thoroughly’ to ‘none’

**What do you do to manage your blood pressure?**

Q4. What actions do you **currently take to manage your blood pressure**? Additionally, what actions have you **previously taken but no longer take to manage your blood pressure**? Please select all the factors that apply to you from the following options.

Q5. Have you ever **forgotten to take your prescribed antihypertensive medication** or **decided not to take them at your own discretion**?

[The following question only applies to those who answered ‘I have forgotten to take my medication in the past’ or ‘I have decided not to take my medication at my own discretion’ in Q5]

Q6. Did you tell your doctor that you **forgot to take your medication** or **did not take your medication at your own discretion**?

[The following question only applies to those who answered other than ‘I told my doctor every time’ in Q6]

Q7. Why did you not tell your doctor about forgetting to take your medication or did not take them at your own discretion?

Please tell us about your **blood pressure**.

Q8. Please tell us your systolic and diastolic **blood pressure a)** **at your first consultation** at a medical institution for the treatment of hypertension; and **b)** **when you first started taking antihypertensive medication**.

Q9. a) What is **your target blood pressure value** and b) what was your **blood pressure values for the past month**?

Where did you measure the blood pressure values entered in a) and b)?

[The following question only applies to those who answered ‘None in particular, I do not know’ for their target blood pressure in Q9]

Q10. Why is [**Systolic blood pressure ● ~ ● mmHg/Diastolic blood pressure ● ~ ● mmHg (Display the target blood pressure values that respondents answered in Q9a)**] your target blood pressure value?

Q11. What kind of explanation did the doctor give you about your target blood pressure? Please provide the numerical target value if your doctor provided you with one

a) Systolic blood pressure
b) Diastolic blood pressure

Please tell us about **blood pressure measurement at home.**

Q12. Do you own a **blood pressure monitor at home**? Please answer even if you do not measure blood pressure at home.

[The following question only applies to those who answered ‘I do not own a home blood pressure monitor’ or ‘I do not know’ in Q12]

Q13. Why do you not own, or do not know whether you own, a home blood pressure monitor?

[The following question only applies to those who answered ‘I own a blood pressure monitor at home’ or ‘I do not know’ in Q12]

Q14. How frequently do you measure your home blood pressure? Please choose the most appropriate out of the following options.

[The following question only applies to those who answered ‘I measure my blood pressure at home’ in Q14]

Q15. When do you measure your blood pressure at home?

[The following question only applies to those who answered ‘I measure my blood pressure at home’ in Q14]

Q16. Why do you continue to measure your home blood pressure?

[The following question only applies to those who answered ‘I measure my blood pressure at home’ in Q14]

Q17. When you measure your home blood pressure, how many attempts do you make to measure your home blood pressure?

[The following question only applies to those who answered ‘I measure my blood pressure at home’ in Q14]

Q18. How do you usually tell your doctor about your home blood pressure measurements? Please choose the most appropriate option from the following options.

[The following question only applies to those who answered ‘I measure my home blood pressure on more than one attempt’ in Q17]

Q19. When you measure your blood pressure on a number of attempts, which value to you record as your home blood pressure?

[The following question only applies to those who answered ‘I do not own a blood pressure monitor at home’ in Q12 or to those who answered ‘I do not measure my home blood pressure’ or ‘I measure my home blood pressure less than once a week’ in Q14]

Q20.

[If Q14 = ‘I measure my home blood pressure less than once a week’]

Why do you measure your home blood pressure less than once a week?

[If Q12 = ‘I do not own a blood pressure monitor at home’ and Q 14 = ‘I do not measure my home blood pressure’]

Why do you not measure your blood pressure at home?

Q21. Have you ever **stopped or reduced the amount or frequency of taking antihypertensive medication at your own discretion**? If so, please provide us with all the reasons from the following options.

Q22. Have you ever **stopped or discontinued visiting the hospital for treatment of hypertension at your own discretion**? If so, please provide us with all the reasons from the following options.
