# Supplementary material for: Physician and patient perspectives on hypertension management and factors associated with lifestyle modifications in Japan: results from an online survey
Source: Hypertens Res. 2020 Jan 29;43(5):450–62. doi: 10.1038/s41440-020-0398-0 (PMC8076050; doi:10.1038/s41440-020-0398-0)
Supplement: Supplementary file 5 — Supplementary Document 5 [file 41440_2020_398_MOESM5_ESM.docx]

**Supplementary Document 5**

**Exclusion Criteria**

Physician survey

Q13:

- Target systolic blood pressure (SBP) value was <100 mmHg or ≥200 mmHg, or
- Target diastolic blood pressure (DBP) value was <60 mmHg or ≥140 mmHg.

Q21 and Q22: The same answer (from seven options available) was selected in response to all 31 factors in *both* Q21 *and* Q22. (Respondent is not excluded if the same answer was given for all questions in *either* Q21 *or* Q22)

Patient survey

SC2: The patient was prescribed ≥30 types of drugs

SC4 and SC7: Same numerical value given for age of patient and the number of years of hypertension treatment received (Respondent is not excluded if there is a possibility that hypertension developed during childhood [as calculated from the difference between the two values])

SC10: Entered ≥90 minutes for duration of initial or follow-up consultation.

Q8:

- SBP was <100 mmHg, or
- DBP was <60 mmHg

Q9:

- Target SBP was <100 mmHg or ≥200 mmHg, or
- Target DBP was <60 mmHg or ≥140 mmHg

Q18 and Q19:

In Q18, answered “I share all my home BP records with my physician” but answered “I do not record my home BP” in Q19.

Q21 and Q5:

- In Q21, answered “I have *never* stopped taking antihypertensive medication or decreased my dosage at my discretion”, but answered “Sometimes I did not take my medication at my own discretion” in Q5.
- In Q21, answered “I sometimes carelessly forgot to take my antihypertensive medication”, but did not select “I sometimes forgot to take my antihypertensive medication” in Q5.
