# Supplementary material for: Physician and patient perspectives on hypertension management and factors associated with lifestyle modifications in Japan: results from an online survey
Source: Hypertens Res. 2020 Jan 29;43(5):450–62. doi: 10.1038/s41440-020-0398-0 (PMC8076050; doi:10.1038/s41440-020-0398-0)
Supplement: Supplementary file 6 — Supplementary Document 6 [file 41440_2020_398_MOESM6_ESM.docx]

**Supplementary Document 6**

**Physicians and patients excluded from analysis**

| Question number | Reasons | n |
| --- | --- | --- |
| **Physicians** |  |  |
| Q13 | Target SBP was <100 mmHg or ≥200 mmHg, or target DBP was <60 mmHg or ≥140 mmHg | 5 |
| Q21, 22 | Selected the same answer (from seven options available) in response to all 31 factors in Q21 and Q22 | 19 |
| Total excluded |  | 24 |
| **Patients** |  |  |
| SC2^a^ | Prescribed ≥30 types of drugs | 2 |
| SC4^b^, SC7^c^ | Years of treatment was equal to age | 1 |
| SC10^d^ | Initial or follow-up consultation lasted ≥90 minutes | 16 |
| Q8 | SBP was <100 mmHg DBP was <60 mmHg | 8 |
| Q9 | Target SBP was <100 mmHg and ≥200 mmHg Target DBP was <60 mmHg and ≥140 mmHg | 29 |
| Q19, Q18 | Answered ‘I do not record my home BP’ in Q19, but answered ‘I share all my home BP records with my physician’ in Q18 | 1 |
| Q5, Q21 | Answered ‘I have never stopped taking antihypertensive medication or decreased my dosage at my own discretion’ in Q21, but answered ‘Sometimes I did not take my medication at my own discretion’ in Q5.  Answered ‘I sometimes carelessly forgot to take my antihypertensive medication’ in Q21, but did not select ‘I sometimes forgot to take my antihypertensive medication’ in Q5. | 37 |
| Additional to the above patient criteria | The age and sex composition of the analysis population was matched to that of patients with hypertension in Japan as outlined in the JSH 2014 guidelines; those who completed the survey after a predefined target proportion for the respective age and gender groups has been met were not included in the analysis. | 201 |
| Total excluded |  | 289 |

All physician and patient questions can be found in Supplementary Table 3 and 4 (physicians) and 5 and 6 (patients) unless otherwise stated.

^a^SC2 patient question: How many medicines are you prescribed in total from clinics/hospitals?
^b^SC4 patient question: What is your current age?
^c^SC7 patient question: How many years have you been attending clinics/hospitals for the treatment of hypertension?
^d^SC10 patient question: How long was your average consultation with your physician at initial consultation (first visit) and the subsequent visits (follow-up or regular visits)?
